# A Fog Computing Solution for Context-Based Privacy Leakage Detection for Android Healthcare Devices

**DOI:** 10.3390/s19051184

**Published:** 2019-03-08

**Authors:** Jingjing Gu, Ruicong Huang, Li Jiang, Gongzhe Qiao, Xiaojiang Du, Mohsen Guizani

**Affiliations:** 1 MIIT Key Laboratory of Pattern Analysis and Machine Intelligence, College of Computer Science and Technology, Nanjing University of Aeronautics and Astronautics, Nanjing 211106, China; huangruicong@nuaa.edu.cn (R.H.); nuaa_jiangli@nuaa.edu.cn (L.J.); 1002351818@alumni.sjtu.edu.cn (G.Q.); 2 Department of Computer and Information Sciences, Temple University, Philadelphia, PA 19122, USA; dux@temple.edu; 3 College of Engineering, Qatar University, Doha 2713, Qatar; mguizani@ieee.org

**Keywords:** privacy leakage detection, intelligent medical service, fog computing, Android, context information

## Abstract

Intelligent medical service system integrates wireless internet of things (WIoT), including medical sensors, wireless communications, and middleware techniques, so as to collect and analyze patients’ data to examine their physical conditions by many personal health devices (PHDs) in real time. However, large amount of malicious codes on the Android system can compromise consumers’ privacy, and further threat the hospital management or even the patients’ health. Furthermore, this sensor-rich system keeps generating large amounts of data and saturates the middleware system. To address these challenges, we propose a fog computing security and privacy protection solution. Specifically, first, we design the security and privacy protection framework based on the fog computing to improve tele-health and tele-medicine infrastructure. Then, we propose a context-based privacy leakage detection method based on the combination of dynamic and static information. Experimental results show that the proposed method can achieve higher detection accuracy and lower energy consumption compared with other state-of-art methods.

## 1. Introduction

Intelligent medical service systems integrate the wireless internet of things (WIoT), such as medical sensors, wireless communications, and middleware techniques to monitor and analyze the patient’s physical health in the form of portable, wearable or body-embedding micro-intelligent personal health devices (PHDs). It can also collect and analyze a large amount of patients’ data by various PHDs to perform the disease diagnosis and prevention both inside and outside hospitals with a flexible doctor-patient communication way. In various PHDs with diverse uses, there are a great quantity of devices with Android installed. With the open-source flexibility, strong content delivery system, and numerous Android’s consumers, Android-based PHDs present significant advantages for both designers and consumers.

However, most intelligent medical devices are vulnerable to external attacks, especially when connected to the network or to different types of custom cloud servers, where malicious attackers are ubiquitous. According to the report of Android malicious apps by the 360 Cyber Security Center in 2016 [1], a cumulative of 14.033 million new samples of malicious programs on Android platforms were intercepted. Things are even worse in the medical field and caused great security concerns. There have been many security accidents caused by hacking of medical equipment or related mobile devices [2,3]. For example, a blackmail software attacked some hospitals both in the USA and Germany [4,5] to invade patient monitors and drug distribution systems. As reported by Kaspersky Lab’s global research and analysis team [6], hackers can easily find wireless devices in hospitals and control the network, or even some PHDs for obtaining the patients’ information. What’s more, a large number of mobile health applications have actively collected users’ sensitive information and sent it to their vendors or other third-party domains over HTTP using plaintext [7], which greatly increases the risk of consumers’ privacy being leaked.

Therefore, how to build a secure intelligent medical service system and protect patient’s privacy still remains a very challenging research issue. There have been some works on privacy leakage detection and privacy protection in the wireless sensor network [8,9,10,11], which are generally considered from three aspects: static analysis, dynamic analysis, and integrated analysis of static and dynamic. (1) Static analysis uses static data flow to analyze the direction of the sensitive data flow in the program with the Android package (APK) file [10,12,13,14]. It could detect efficiently with high code coverage, but is not applicable to the analysis of apps with multi-thread methods. (2) Dynamic analysis, on the contrary, could avoid the shortcomings of static analysis when monitoring the running state of software [11,15,16,17,18]. It compensates for static analysis in detection accuracy, but costs much more code coverage, and often lags behind leakage events during the detection. (3) Integrated analysis combines static and dynamic analysis [19], which consists of software piling, automated testing, and protective systems. By the integrated analysis, monitoring codes are inserted through static code piling to obtain data flow information and sensitive application programming interfaces (APIs) usage data. Then the repackaged software is automatically tested and a protective layer is provided to protect devices from malicious software attacks [20].

Our proposal is motivated by such the integration of static and dynamic analysis. However, most of traditional privacy protection methods are unsuitable because PHDs based on WIoT need a strong technological foundation for their rapid development from both the hospitals and patients. Therefore, in this paper, we develop a novel context-based privacy leakage detection method, which is based on an invented fog computing solution [21] for Android PHDs and services. Specifically, first, we design a privacy protection framework for intelligent medical service systems based on fog computing. In this framework, we can monitor privacy leakage of PHDs with the Android system in real time, and process user’s privacy data and the real-time operation status at the fog. Second, we propose an privacy leakage detection method based on Android application by utilizing the context information (described in Section 4). The proposed method combines the static stain analysis with the dynamic hook monitoring, which could effectively detect privacy leakage and provide protection. The experimental results show that our method can achieve higher detection accuracy and lower energy consumption compared with other state-of-art ones.

## 2. Related Work

### 2.1. Intelligent Medicine and Fog Computing

Intelligent medical service and fog computing are hot research topics recently. For example, Ref. [22] proposed an architecture of personalized medical service based on fog computing, and optimized it by the clustering method. Ref. [23] proposed a method of combining drivers’ mHealth data and vehicular data for improving the vehicle safety system to solve the problem of road accidents and incidents, due to various factors, in particular the health conditions of the driver. To deal with the increasing false alarms in frequently changing activities, Ref. [24] presented a user-feedback system for use in activity recognition, which improved alarm accuracy and helped sensors to reduce the frequency of transactions and transmissions in wireless body area networks. Ref. [25] addressed some threats of mHealth networks and focused on the security provisioning of the communication path between the patient terminal and the monitoring devices. To solve the problem of response delay and resources waste in the case of increasing complexity, Ref. [26] put forward a fog-based cloud model for time-sensitive medical applications. Ref. [27] designed a medical and health system based on fog aided computing, which classified user infection categories by decision tree and generates diagnostic alerts in fog layer. To diagnose and prevent the outbreak of Chikungunya virus, Ref. [28] put forward a medical and healthcare privacy data protection scheme based on fog calculation. Ref. [29] proposed a multilevel architecture based on fog computing. Ref. [21] suggested that fog computing will be widely used in intelligent medicine in the future.

### 2.2. Security Based on Android Platforms

Security issues have always been the focus of network research [30,31,32,33]. With the popularity of Android medical equipment and the emergence of malicious software, the privacy protection of Android platform has caused widespread concern in the academic field in recent years. Generally, the research of privacy leakage detection is considered from three aspects: static, dynamic, and integrated analysis of static and dynamic. Static analysis analyzes the APK file, and uses static data flow to analyze the direction of the static sensitive data flow in the program. For instance, Ref. [12] proposed a static privacy leakage analysis system, which first created the mapping between API functions and required permissions. Refs. [10,13,14] used inter-application interactions in Android to mark the components in security. By the static analysis, privacy leakage detection of Android had high code coverage of the software. However, static analyses are incapable of analyzing apps with reflection, multi-threaded, or reference methods. Since static analysis cannot obtain the running state of the softwares, its accuracy may be unsatisfied. Dynamic analysis can avoid such a shortcomings when monitoring the running state of a software. Ref. [15] designed the TaintDroid to perform a dynamic analysis. Ref. [11] performed a dynamic stain analysis of the running mode defaulted by Google in Android 5.0 and the above systems. Similar methods, namely to detect privacy leaks by modifying system codes are DroidBox [16], Mobile-Sandbox [17,34], VetDroid [35], AppFence [36], FlaskDroid [37]. Ref. [38] proposed a privacy leakage monitoring system to repackage the software and insert the monitoring logic codes. Similar systems are AppGuard [39] and Uranine [18]. But detection results of the dynamic analysis possibly lagged behind leakage events [40]. Therefore, some works combined static and dynamic analysis [19,20]. For instance, AspectDroid [19] inserted monitoring codes through static bytecode instrumentation to automatically test the repackaged software, and added a protective layer to protect the device from malicious software attacks. AppIntent [20] combined static data flow analysis and dynamic symbolic execution to verify privacy disclosure, which reduced the search space without sacrificing code coverage.

The works above can solve the problem of privacy leakage to a certain extent, but there are still some shortcomings, as follows: (1) static analysis is unable to get the dynamic running information of the software. Many malicious apps can download executable codes to avoid from the static detection; (2) dynamic analysis usually sacrifices code coverage, and some methods require modification of the source codes of Android systems, which increases difficulty of development at the expense of some system resource; (3) analysis based on app repackaging has some impact on the original app, and some apps are resistant to these methods by using encrypted packers.

## 3. The Privacy Leakage Detection Framework Based on Fog Computing

In this section, we propose a fog computing framework for privacy leakage detection of healthcare networks to protect the intelligent medical service systems. Basically, it monitors various applications on PHDs in real time, detects malicious codes, and feeds detection results back to users. Moreover, this framework is combined with fog computing to conduct encryption, decryption, and identity authentication of the user’s privacy data.

### 3.1. Intelligent Personal Health Devices

The applications of intelligent PHDs can be divided into two parts.

**Figure 1:**
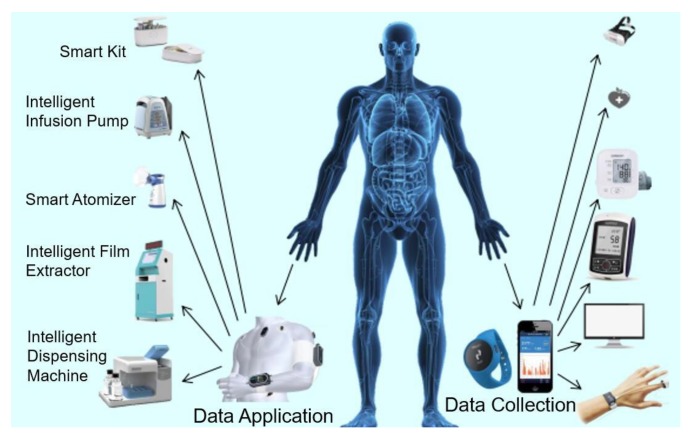
Healthcare devices.

For data collection, PHDs collect data through diverse sensors on users and upload data to a healthcare monitoring and management center, so as to perform 24-h health monitoring. PHDs collect and send users’ physiological health data, such as electrocardiography (ECG), heart rate, and blood pressure. Figure 1 is a diagram of commonly used PHDs, which fall into three categories: (1) portable small medical devices, or micro-intelligent chip devices which can be worn on or embedded into the human body; (2) indoor care devices, such as smart medicine boxes; (3) medical equipment used in hospitals, such as intelligent film extractors.

For data application, the collected data is sent to the fog for customizing different treatments to users. For example, smart medicine box reminds the user to take medicine on time. The intelligent infusion pump adjusts the infusion rate by observing the changes of blood pressure and other information. The intelligent atomizer can revise the atomization time and dose according to the body condition. The intelligent film taker reduces the queue waiting time, and outputs corresponding medical images based on patients’ biological characteristics. The intelligent dispenser can precisely configure user-defined drugs.

### 3.2. System Architecture

**Figure 2:**
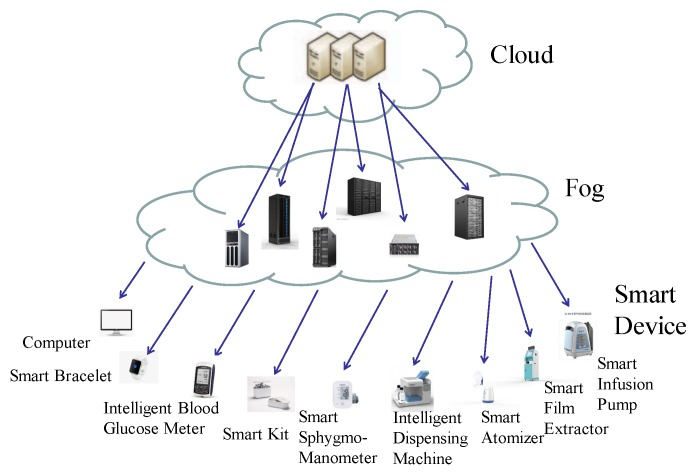
Three-layer architecture.

In this paper, we propose a privacy leakage detection method based on fog computing framework, which is a higy vitalized platform that provides computing, storage, and network services. The architecture of the designed system here is shown in Figure 2, which shows the components and interrelationships, namely cloud, fog interface and intelligent medical terminal. Generally, fog nodes work between terminal devices and traditional cloud computing data centers, which means that they are physically closer to the users. Besides, fog has less requirements of network bandwidth, so it reduces the network costs and time delay. Due to these features, fog not only extends the capabilities of the cloud, but also reduces the requirements for the organization to apply it.

As illustrated in the Figure 2, the cloud manages the data storage, computing, and information processing, while the fog mainly provides computing and storage resources for the lower level, including making the information of upper and lower levels inter-operable, data analysis and management, and security protection. As the services could require excessive computing and storage resources beyond the capacity of the fog, the cloud will provide a replacement service at this situation. In the lower level, terminal devices carry out the collection of data, transmission and simple processing of information, and so on.

**Figure 3:**
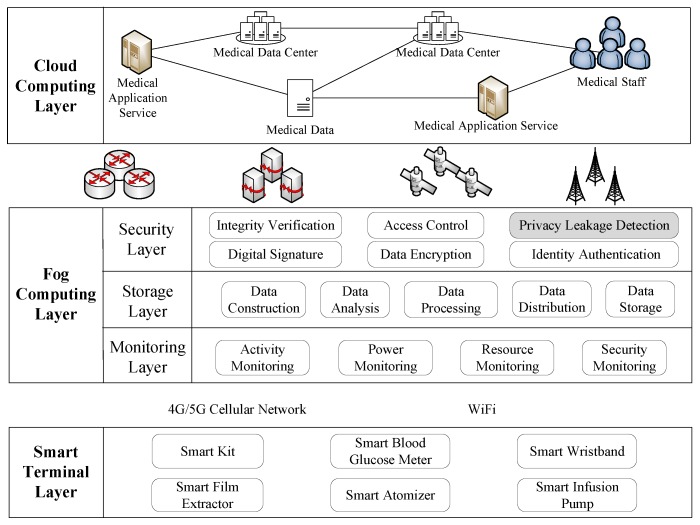
Logical architecture of fog computing for the healthcare network.

Figure 3 is the logical architecture of fog computing for the healthcare network. The first layer is the intelligent PHDs layer, including smart wristbands, smart blood glucose meters, smart film extractors, etc. They are mainly used for collecting health information of users, and sending the information to the fog computing layer for the further processing.

The second layer is the fog computing layer, composed of three sub-layers: monitoring layer, data storage layer, and security protection layer. The monitoring layer includes activity’s monitoring, power status monitoring, resource monitoring, and service monitoring. In this sub-layer, monitoring information is sent to users and abnormal information is detected. In the data storage layer, data received from the intelligent PHDs layer are filtered and pruned for data analysis to extract the privacy information. In the security protection layer, there are integrity verification, access control, digital signature, data encryption, identity authentication and privacy leakage detection which is the main issue in this paper. Specifically, based on fog computing, we design an Android malicious code monitoring scheme to prevent intrusion by illegal users and dynamic malware monitoring of various applications on devices.

The third layer is the medical cloud computing layer for processing, storing and generating user personal files.

### 3.3. Privacy Leakage Detection

In this paper, we design a privacy leakage detection method with the combination of the fog and users due to the strong capabilities of data processing and network control of the fog. It is mainly for intelligent PHDs based on Android systems, and protects the user’s private information by monitoring in real time.

**Figure 4:**
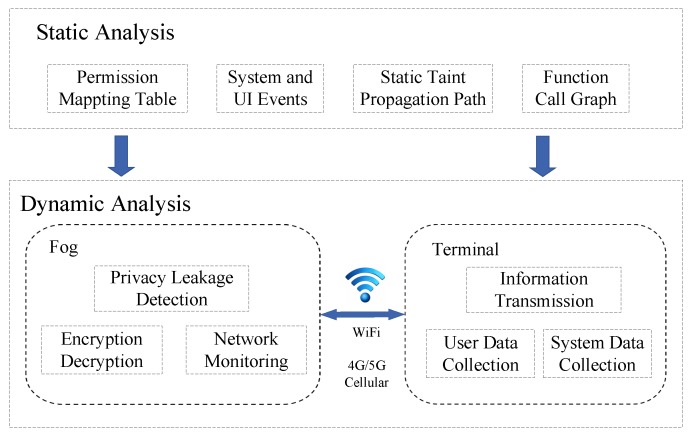
Framework of privacy leakage detection.

Basically, we use context analysis technology to design the detection scheme, including static privacy leakage analysis and dynamic privacy disclosure monitoring, as shown in Figure 4. First, we use static analysis to analyze the permissions mapping to various API functions, system and user interface events, static taint propagation path, and function calls. Next, we perform dynamic privacy leakage monitoring, which mainly includes the following four stages: (1) the users’ information and system working status are collected by PHDs at the user terminal for constructing the context information and transmitting to the fog; (2) on the fog, the privacy data is extracted for encryption, and the monitoring data collected in the user terminal is analyzed for performing the privacy leakage detection by the access control technology (Section 4.2); (3) if a privacy leakage is detected, the next data-transmission will be blocked. Meanwhile, the fog will intercept the behavior of the privacy leakage and notify the user for protecting the user’s information; (4) the fog uploads the user information and the system status to the cloud periodically.

Note that, for the convenience, in this paper we use MyPrivacy to present the collection mechanism of the privacy and system information on the user terminal, and FogPrivacy to present the privacy protection mechanism (such as privacy leakage detection) on the fog.

## 4. Context-Based Privacy Leakage Detection

Our context-based privacy leakage detection method includes two parts: static privacy leakage analysis and dynamic privacy leakage monitoring, which are carried out by the combination of the fog and the user terminal. The static analysis constructs the context of the privacy-related API function to predict trigger events and the possible privacy leakage of the API call. Dynamic monitoring intercepts privacy API by using hook technology to predict the privacy leaks which may be caused by API calls. If there is a privacy leakage, it will be automatically blocked.

### 4.1. Static Privacy Leakage Analysis

**Figure 5:**
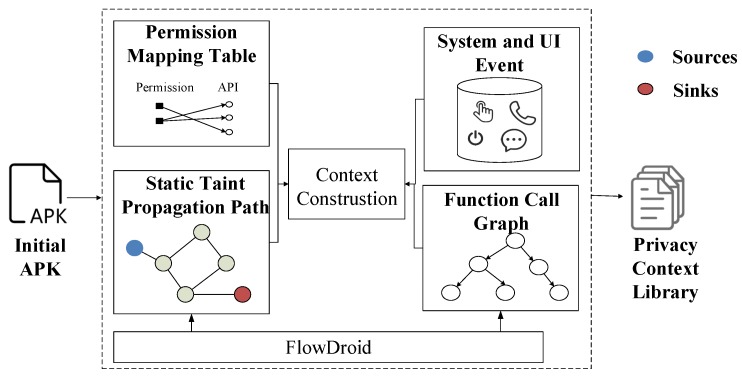
Frame of static privacy leakage analysis mechanism.

The static analysis is used to construct the context of the software privacy-related API functions, which is based on the FlowDroid [41]. From the static analysis, the path between sources and sinks can be found, and the sequence of sensitive function calls could be extracted. The framework of the static privacy leakage analysis mechanism is shown in Figure 5, which contains five parts:

(1) Static taint propagation path: it inputs the original APK application installation package, and configure the sources and sinks function files. Then the system performs the static stain analysis through the FlowDroid platform and finds out the possible privacy leakage path. Here, we use the data of the Susi project [42] to mark the sources and sinks functions for increasing the coverage of the privacy functions.
(2) Function call graph: it extracts the Java codes from the decompiler APK, then constructs the function call graph using Soot [43], a Java language optimization framework.
(3) Permission mapping table: it generates the permission-API mapping table based on the PScout project [44], which describes the relationship between an API and its corresponding permissions by scanning the Android code and has a more accurate result than the Google’s official API.
(4) System and UI event: it analyzes privacy entry functions and triggering conditions from a large amount of system and UI event information. Here, the system events include various callback functions of Android system (such as receiving text messages and changing network state), as well as the lifecycle functions of Android components (onCreate, onRestart, etc.). The UI events contain the user’s interaction with the software interface (such as clicking a button, pressing the volume key).
(5) Context construction: it builds the corresponding context information based on Algorithm 1.

Here, we present the definitions and description of Algorithm 1 as follows.

**Definition** **1.** *A function call graph is a directed graph CG=(N,E), where N represents the set of functions in the software and E is the set of edges. For example, e(a,b)∈E represents that the function a calls function b.*

**Definition** **2.** *In a path $p_{s2s}$ from source to sink, $p_{s2s}=n_{source}n_{1}n_{2}\ldots n_{sink}$, where $n_{i}\in N(i=source,$1,2,…,sink) are called a privacy leakage path.*

**Definition** **3.** *In a function call graph CG=(N,E), if there is a path $p=n_{e}n_{1}n_{2}\ldots n_{source}$, and there are no edges that go into $n_{e}$ ($\forall n\in N,e(n,n_{e})\notin E$), then $n_{e}$ is called a privacy entry point, which means that no functions in N call $n_{e}$. Since Android is an event-driven operating system and its components used for development have their own lifecycles, the privacy entry point functions generally consist of various message response functions and lifecycle functions.*

**Definition** **4.** *A privacy API function context information PrivacyContext is a triple shown in Equation (1),*

(1) PrivacyContext=(api,permission,context),

where ‘api’ represents the name of the privacy-related function. The set ‘permissions’ is the set of permissions that the privacy-related function requires. context is the set of $<p_{s2s},n_{e}>$ pairs, where each pair contains a privacy leakage path $p_{s2s}$ and its corresponding entry point function $n_{e}$.

**Algorithm 1** Context construction algorithm.

**Input**
 Function Call Graph CG Privacy Disclosure Path Paths System and UI Events Events Permission Mapping Table Table
**Output**
 Context PrivacyContext
**Begin**
 PrivacyContext=null context=null **for all** e∈E **do**  **if** e.target∈PrivacyAPI **then**   $n_{e}=getEntryPointFromCG\left(e.origin\right)$ //Retrieval entry function from CG   permission=getPermissionFromTable(e.target) //Retrieval API permission   **for all** path∈Paths **do**    **if** path.source==e.target **then**     $context.add(<path,n_{e}>)$ //Add context    **end if**   **end for**   PrivacyContext.add(e.target,permission,context)   context.clear()  **end if** **end for** **return** PrivacyContext


The main idea of the Algorithm 1 includes: (1) traverse each edge from the function call graph CG, and locate the privacy-related function and its corresponding permission using the permission mapping table; (2) get the privacy entry point functions for the API call from the function call graph. Privacy entry point functions are defined in Definition 2; (3) for a chosen edge, find out all subsequent edges in static taint propagation path as possible privacy leakage path for the API call, and generate the context information for the privacy-related API function. After context construction, the context information PrivacyContext is loaded into the privacy context library, which will be deployed on the fog.

Here, we list the general categories of the privacy data in the system of PHDs in Table 1, including device resource, system information, login data and user data. Here, device resource represents the information of devices, which depends on the specific input from external devices (e.g., GPS). Its privacy-related API functions are getLatitude() and getLongitude(). System information describes attributes and labels of the device systems (e.g., international mobile equipment identity (IMEI)). Its privacy-related API function is getDeviceId(). Login data is the login data entered by the user, which mainly includes the account password. Its privacy-related API function is getPasswd(). User data is user-related information, such as step count, heart beat and sleep status, with the privacy-related API functions stepListener, heartListener and sleepListener respectively.

### 4.2. Dynamic Privacy Leakage Monitoring

**Figure 6:**
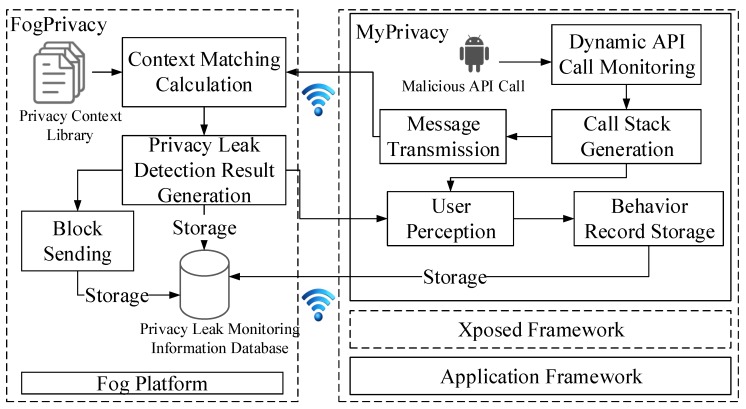
Dynamic privacy leakage monitoring.

Static analysis cannot reflect the real state of the app. Besides, some malicious apps can download malicious third party libraries and executable programs and execute them dynamically to steal privacy information. Static analysis can not detect this kind of attack efficiently. Therefore, we proposed a dynamic monitoring scheme for privacy leakage based on fog computing, which is realized by the combination of the fog and user terminal. On the user terminal, dynamic behaviors of the app are monitored by the key privacy-related API function of dynamic hook technology. Then the real state of the app is obtained and the relevant information is sent to the fog. At the fog, similarity between the static analysis results and the dynamic behavior information is calculated to find out the possible privacy leakage risks of the dynamic API calling behavior. Finally, the result of similarity comparison is sent back to the user terminal and the software behavior which poses a risk of privacy leakage would be blocked and informed back to the user. The framework of the proposed dynamic monitoring mechanism is shown in Figure 6.

The main modules in the Figure 6 have the following functions:

(1) Dynamic API call monitoring: dynamic API call monitoring module uses Xposed-based hook technology to write privacy-related API monitoring code. By collecting API function call stack information, the dynamic API execution context information is constructed and sent to the fog.
(2) Context matching calculation: on the fog, the context information database of privacy API functions derived from the static analysis is matched with the context information of API dynamic execution. The matching calculation algorithm is shown in detail below.
(3) User perception: when the detection result of the fog indicates that the suspicious call may cause privacy leakage, the fog will send the detection results to the user terminal. Through the user perception module, the event information that triggers the API and the risk of possible privacy leakages will be prompted to the user. The system intercepts the invocation of the related API and blocks the privacy leakage.
(4) Behavioral log module: the behavioral log module makes quick judgments about similar situations during follow-up monitoring. The module would format and store the information of each API call and the user’s choice. Then it feeds the information back to the fog, and the fog will store these information in the privacy leakage monitoring information database.
(5) Privacy leakage monitoring information database: the privacy leakage monitoring information database keeps information on privacy leakage monitoring of all PHDs on the fog and uploads it regularly to the cloud for permanent preservation.

The context matching algorithm (Algorithm 2) and its related definitions are introduced below.

**Algorithm 2** Dynamic context matching algorithm.

**Input**
 Context DynamicContext,PrivacyContext
**Output**
 The closest pc of the API call
**Begin**
 similarity=0 result=null **for all** pc∈PrivacyContext **do**  **if** pc.api==DynamicContext.api **then**   simTemp=Similarity(DynamicContext.stack,pc.context) //Calculate similarity   **if** similarity<simTemp **then**    similarity=simTemp    result=pc// Update result   **else if** similarity==simTemp **then**    result.add(pc)// Add result   **end if**  **end if** **end for** **return** result

**Definition** **5.** *The execution context information of a dynamic API (DynamicContext) represents the api and call stack information, as shown in expression (2),*

(2) DynamicContext=(api,stack<funcs>)

where api represents the system function api calls. stack<funcs> represents the call stack information of the function.

**Definition** **6.** *Given an API dynamic execution context information DynamicContext=(api,$<f_{1},f_{2},\ldots ,f_{n}>)$ and a call path $p=n_{1}n_{2}\ldots n_{m}$ in the static function call graph CG, the similarity between them is calculated according to Equations (3) and (4).*

(3) $$Similarity=\frac{\sum_{i=1}^{n}\sum_{j=1}^{m}F\left(f_{i},n_{j}\right)}{n}$$

(4) $$F\left(f_{i},n_{j}\right)=\left\{\begin{array}{cc} 1, & f_{i}=n_{j}\\ 0, & f_{i}\neq n_{j} \end{array}\right.\left(1\leq i\leq n,1\leq j\leq m\right),$$

where *n* is the length of the function call stack in DynamicContext, that is, the number of functions in the function call stack. *m* is the length of the call path *p*. Function *F* is used to identify whether two functions for similarity calculation are equal.

From the Equations (3) and (4), the similarity is calculated. Then, we can get the context information which is closest to the API function call from Algorithm 2. With the information, it is possible to predict the privacy disclosure that may occur when the API is called. We extract Android system events from the dynamic execution context information of the API, which directly lead to the API call. When a privacy leakage occurs, calls are blocked and the leakage is prompted for privacy protection.

## 5. Experimental Verification and Results Analysis

In order to verify the effectiveness of the proposed method, we conducted two kinds of experiments, i.e., the static analysis experiment and the dynamic monitoring experiment. We used a testing PC machine (CPU: Intel Core i5-6500, RAM: 8GB, OS: 64-bit Ubuntu 16.04) as a fog node and a *Samsung GT-I9500, Samsung Electronics Co., Ltd., Korea* (https://www.samsung.com) (Android 4.4.4 system with Xposed) to conduct the experiments. The dataset consisted of 397 malicious samples and 300 benign samples. The malicious samples were selected from the DREBIN dataset [45,46], and the benign ones included 10 types of apps, all of which are downloaded from Google Store and China Mall. We use this dataset to verify the algorithm and the reliability of the proposed scheme.

### 5.1. Privacy Leakage Monitoring Experiments

**Figure 7:**
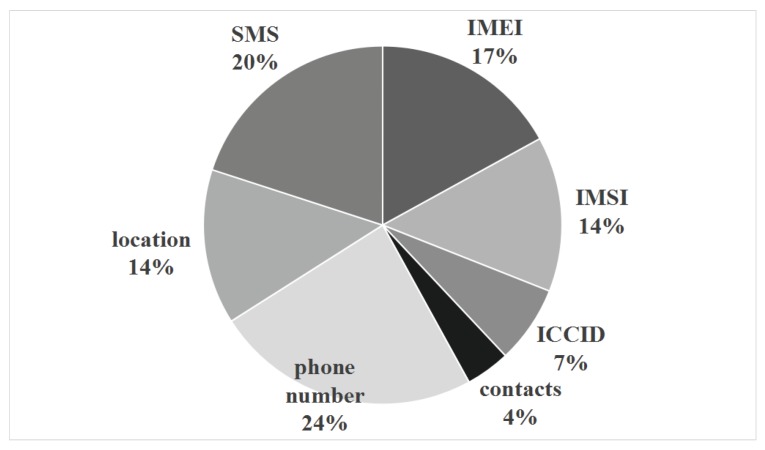
Proportion of privacy leakage types.

First, we performed the static privacy leakage detection experiment. We constructed the context information of the privacy-related API function of the software. In our experiments, we considered on following types of privacy: international mobile equipment identity (IMEI), international mobile subscriber identification number (IMSI), integrated circuit card identity (ICCID), short messaging service (SMS), contacts, phone number, and location. For leakage events, we focus on the network transmissions, logs and SMS messages. From the context information, we can obtain kinds and proportions of the privacy data, as shown in Figure 7.

From the Figure 7, we can find that leakage of phone number is the most common, reaching about one quarter (24%), followed by short messages (20%), IMEI (17%), IMSI (14%) and location (14%).

From the privacy-related API context information, we also can get the following data, as shown in Table 2 by counting the data of each privacy entry.

Table 2 shows that the privacy entry point functions dominated by the lifecycle functions (described in the Section 4.1). For several apps (package name: com.gp.lights and com.keji.danti607), the static analysis of them finds that, in order to disguise themselves, malware actions often occur when the status of Android components change. Privacy leakages that happened on the first run of the app were rarely seen, for reducing the probability of being discovered by users.

Furthermore, we installed the test application on the real machine, and built the Xposed framework with the coded MyPrivacy and the FogPrivacy program, which includes:

(1) Detection platform: in order to compare the accuracy of our method, we used the test results of DroidBox platform as a baseline, which modifies Android systems based on TaintDroid [15] and has extra functions of stain analysis and call monitoring. With its output (the log), we can analyze the detection results.
(2) Behavior triggers: Generally, malware actions (such as privacy theft) were set to be triggered under certain conditions for hiding their sensitive behavior. This type of malware makes function calls by tapping of system events, which is declared in the AndroidManifest.xml file of all tapping events in Android. Thus we decompiled the APK file, and extracted the tapping events to be stored in the the database.
(3) Result analysis: We used manual de-compilation to analyze the results by using JEB2 [47] to find out the reason that caused the differences.

We compared results of two platforms, as shown in Table 3. From the table, we can observe that MyPrivacy detects more privacy leakages than DroidBox. MyPrivacy had 2876 pop-up windows and DroidBox had 2431 MyPrivacy leaks in its log records. There are 1780 same leakage events in the same operation, which means that both detection platforms successfully detected the same 1780 leakages. After analyzing the results and manual de-compiling of the software, we found that DroidBox run the tests with an emulator, which could be detected by some malwares through IMEI number, telephone number and other information. As a consequence, some malicious codes could not successfully triggered. MyPrivacy, however, is installed in a real mobile phone, making this type of privacy to be detected.

### 5.2. Comparison of Experimental Results

As *hook* technology used in the proposed method, we tested the system performance before and after the installation of MyPrivacy on the Antutu Benchmark, Quadrant Benchmark respectively, on the Android phones (*Samsung GT-I9500, Samsung Electronics Co., Ltd., Korea*, Google Nexus (www.google.com/nexus/), *Xiaomi M6, Xiaomi Technology Co., Ltd., China* (https://www.mi.com/global/mi6/) and *Huawei STF-AL10, Huawei Technologies Co., Ltd., China* (https://www.huawei.com/cn/)). The results are shown in Table 4.

In Table 4, “*before*” and “*after*” mean the benchmark results of evaluating the performance of each hardware before and after MyPrivacy is installed on a device. According to the evaluation results, MyPrivacy (FogPrivacy) caused a little extra energy consumption (no more than 5%) on all phone systems and platforms, which was within the allowable range.

Table 5 shows the comparison results between our method and some other privacy leakage detection methods on the Android system: LeakMiner [15], FlowDroid [11], TaintDroid [35] and Aurasium [41]. Since most of the comparative methods do not provide test data and system source codes, we conducted the comparison from the perspective of the analytic methods and features, i.e., whether customized system was needed, whether modification of the application itself was needed and whether this method was able to prevent the leakage.

In the systems shown in Table 5, the methods of privacy leakage detection based on different analysis and detection strategies (i.e., features) were selected to perform the detection. LeakMiner used the static function call graph as the basic analysis data, by calling the reachable relationship of the marked function in the graph to determine whether there is a privacy leakage. The method was simple to practice, and with high code coverage. Similarly, FlowDroid used static taint analysis, taking Android lifecycle functions into consideration. These two systems did not need to modify the app or Android system. However, they were both unable to prevent leakages when the app was running due to the shortcomings of static analysis, i.e., offline analysis. TaintDroid and Aurasium were two privacy leakage detection schemes based on dynamic analysis. TaintDroid modified the system and inserted taint analysis code, and Aurasium repackaged the software itself to add privacy disclosure decision logic. Both of them can carried out real-time privacy data usage monitoring. However, TaintDroid can just conduct privacy leakage reports in the form of system notifications, and it modifies the system codes, making it less adaptive. Aurasium allows users to intercept the leakage, but repackaging may affect the app, or may fail if the app uses some reinforcement methods. In this experiment, our method consistently performed the best in all conditions, due to the combination of the static and dynamic analysis, which ensures the code coverage and the real-time performance. As *hook* technology is non-intrusive to Android system, our method could guarantee detection without sacrificing adaptability.

### 5.3. Security Experiment of Smart Wristband App

**Figure 8:**
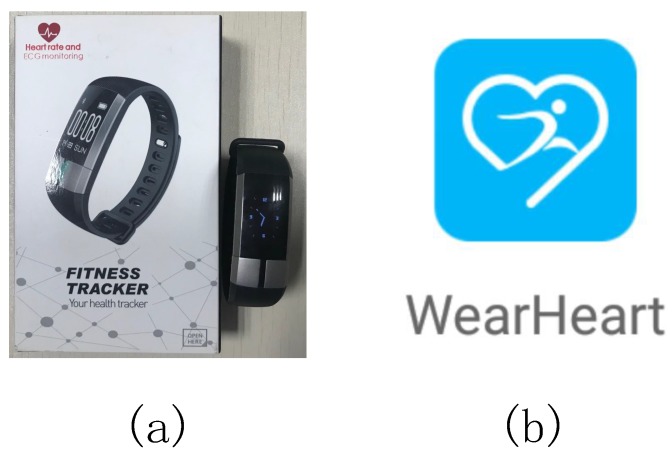
*Example* of a smart wristband. (**a**) The wristband. (**b**) App of clients.

**Figure 9:**
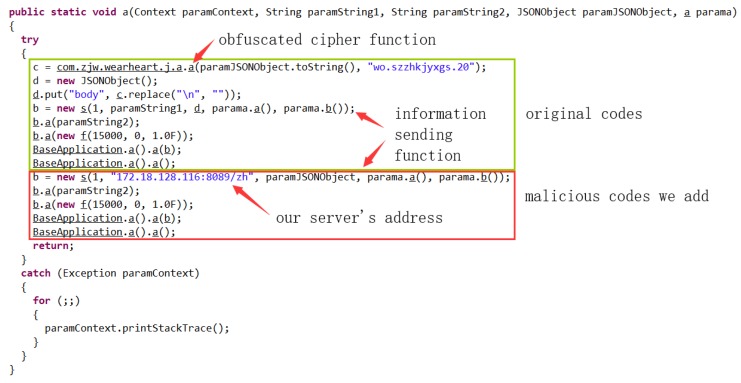
Malicious codes in Java form.

**Figure 10:**
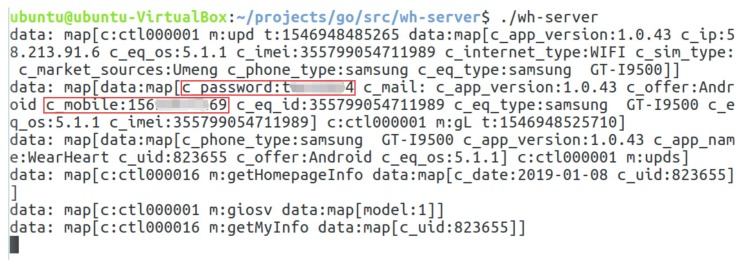
Decoded information received from the malicious application.

**Figure 11:**
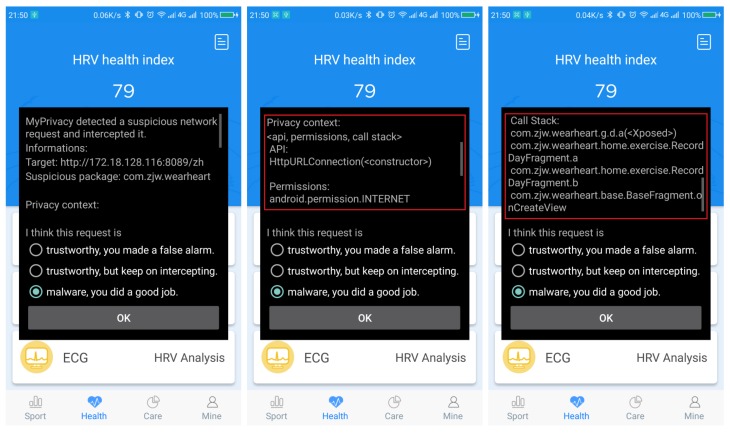
Interception to the malicious app.

To further verify the validity of our method, we conducted a vulnerability analysis of a smart wristband application to find possible privacy leakage problems. The wristband and its app are shown in Figure 8. The experiment was carried out as follows:

(1) Vulnerability analysis: we used FlowDroid to perform static analysis of the original app to verify its vulnerability. We did find out some vulnerabilities that might be exploited. For instance, the app uses hard-coded URL, data transmission by HyperText transfer (HTTP) protocol without encryption, the “send data” function follows immediately after a Java built-in AES cipher function.
(2) Malicious code insertion: based on the above analysis in (1), we found that the app would send user data to a specified URL. If the specified URL was modified maliciously to be the server address of a hacker, the user’s privacy data would be obtained constantly. To simulate such an attack, we used Apktool, an apk analysis tool to unpack the app, and some malicious codes were inserted in the unpacked smali file. Specifically, first, we searched for functions that use the hard-coded URL as a parameter, because those functions may call network transmission APIs and send the information to this address. Then, we modified the parameter to our server address and call those functions again. The malicious code copies the user data and sends it to the server we experimented with. Figure 9 shows the malicious codes in detail.
(3) Privacy leakage detection and display: the malicious code inserted in (2) would divulge user’s privacy, including a user’s login name and password, and health data. Leaked data is illustrated in Figure 10. Some sensitive data are marked in red. As can be seen from the figure, login password and the phone number was disclosed in plaintext.
(4) Detection and interception test: MyPrivacy focused on accessing the dynamic environment and running information of the user-interface. FogPrivacy was responsible for privacy leakage detection and interception. When a running application attempts to send information over the network, the user will be informed of the location to which the data would be sent to determine target trustworthiness. If the request of an app was rejected by most users, FogPrivacy and MyPrivacy would mark it as a malicious behaviour and send the rejection record to the user-interface. From Figure 11, we can see that MyPrivacy poped up a window, because FogPrivacy had successfully intercepted the suspicious network transmission. The context information is also shown in the pop-up window, which is in the red rectangle.
  In this case, the complete context is:
  API
  HttpURLConnection(<constructor>)
  Permissions
  android.permission.INTERNET
  CallStack
  com.zjw.wearheart.g.d.a(<Xposed>)
  com.zjw.wearheart.home.exercise.RecordDayFragment.a
  com.zjw.wearheart.home.exercise.RecordDayFragment.b
  com.zjw.wearheart.base.BaseFragment.onCreateView
(5) Discussion: this attack is a kind of app piggybacking (repackaging) attack, and should have been the meal of research [48,49]. However, the original app (benign one) does not appear in most popular app markets, so most detection systems have little knowledge of this app at the beginning (especially signature information). When they analyze this pair of apps, they could hardly tell which app is benign or malicious. We once uploaded the original app and the maliciously modified one to VirusTotal, a malware analysis website, for detection, and found that both applications passed all security tests. In this situation, wristband users could be easily cheated to install a compromised app, if it is uploaded to app markets.

## 6. Conclusions

In this paper, we studied the privacy protection method of PHDs based on fog computing. We proposed a framework for security and privacy protection based on fog computing in IoT healthcare networks. We analyzed the internal mechanism of software accessing private data, presented the method of constructing the context information base of privacy-related API functions and proposed a new method of privacy leakage detection method. Experiment results showed that our method had a efficient detection of privacy leakage and outperformed state-of-the-art methods.

## Figures and Tables

**Table 1:** Some sensitive privacy-related API functions.

Classification	Example	API
Device Resource	GPS	getLatitude, getLongitude
System Infomation	IMEI	getDeviceId
Login Data	Password	getPasswd
User Data	StepCount	stepListener
User Data	SleepStatus	sleepListener
User Data	Heartbeat	heartListener

**Table 2:** Entry point statistics for privacy leakages.

Entrypoint	Lifecycle Method	System Event	UI Event
Proportion	84.5%	9%	6.5%

**Table 3:** Accuracy comparison.

Platform	Total Leakage Count	Same Results
MyPrivacy(FogPrivacy)	2876	1780
DroidBox	2431	

**Table 4:** System performance.

Platform	Before	After	Phone Type	Extra Energy Consumption
AnTuTu	56,333	53,629	SamSung SM-N900	4.8%
	40,479	39,186	Google Nexus 5	3.19%
	55,186	52,923	Xiaomi MI6	4.10%
	68,357	65,212	Huawei STF-AL10	4.60%
Quadrant	49430	47670	Samsung SM-N900	3.56%
	36,320	34,849	Google Nexus 5	4.05%
	39,035	37,559	Xiaomi MI6	3.78%
	39,882	38,729	Huawei STF-AL10	2.89%

**Table 5:** Comparison with other systems.

System	Method Type	Feature	Customized System Is Needed	Modification of Application Is Needed	Able to Prevent Leakages
LeakMiner	Static Analysis	Function Call Graph	No	No	No
FlowDroid	Static Analysis	Static Taint Analysis, etc.	No	No	No
TaintDroid	Dynamic Analysis	Dynamic Analysis, etc.	Yes	No	No
Aurasium	Dynamic Analysis	App Repackaging	No	Yes	Yes
Our Method	Static and Dynamic Analysis	Function Call Graph, Dynamic Analysis, etc.	No	No	Yes
